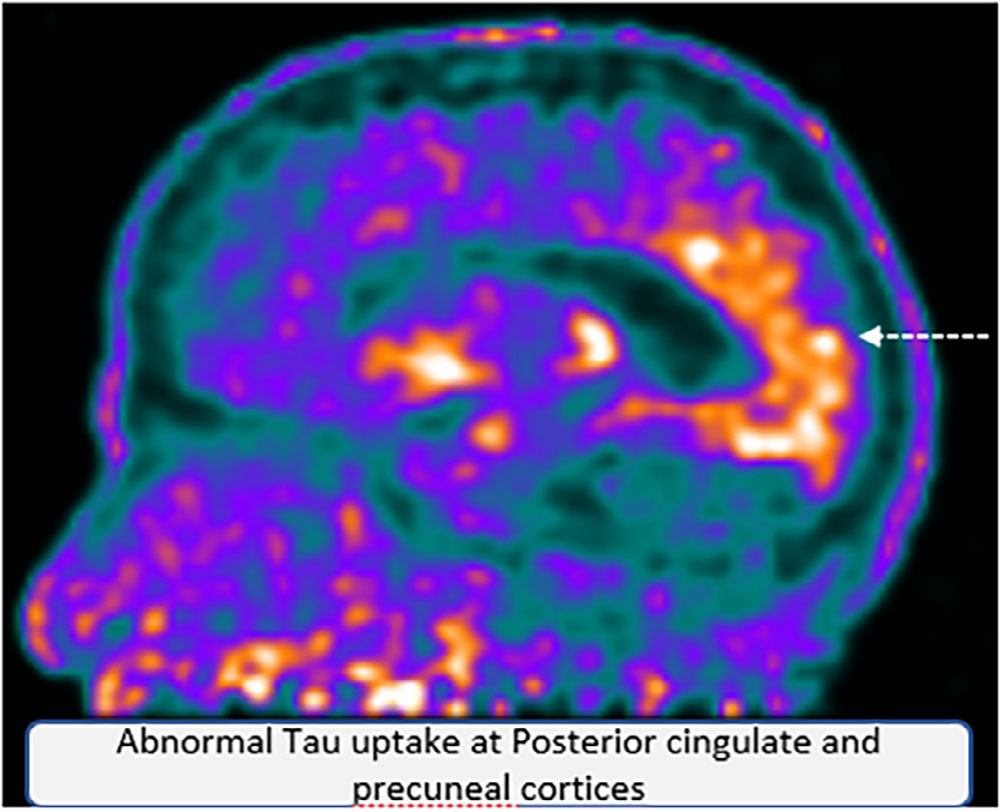# Unravelling Posterior Cortical Atrophy: Clinico‐Imaging Insights from a Tertiary Care Cohort in India

**DOI:** 10.1002/alz70857_102003

**Published:** 2025-12-24

**Authors:** Anu Gupta, Arnab Adhya, Anjali Anjali, Aditi Dubey, Madhavi Tripathi, Roopa Rajan, Ajay Garg, Mamta Bhushan Singh, Venugopalan Y Vishnu, Rohit Bhatia, Achal K Srivastava, Manjari Tripathi

**Affiliations:** ^1^ All India Institute of Medical Sciences, New Delhi, Delhi, India

## Abstract

**Background:**

Posterior Cortical Atrophy (PCA), first described by Benson et al., is a progressive neurodegenerative disorder affecting visual and posterior cognitive functions, while sparing memory and language in early stages. Although Alzheimer's Disease (AD) pathology underlies most cases, alternative etiologies like Dementia with Lewy Bodies (DLB), Corticobasal Degeneration (CBD), and Prion disease have also been reported. This study examines the clinical, etiological, and imaging profiles of PCA patients evaluated at a tertiary care center in India, using Crutch et al. 2017 consensus criteria.

**Method:**

Eight PCA patients diagnosed at a tertiary hospital in India between 2021 and 2024 were included in this prospective case series. Demographic data, neuropsychological profiles, imaging findings, and biomarker studies were analyzed. Patients were assessed for visual and posterior cognitive impairments.

**Result:**

PCA predominantly presented as early‐onset cognitive decline in the 5th to 6th decade, with equal gender distribution. All patients exhibited initial visuospatial, visuoperceptual, or praxis impairments. Based on Crutch et al.'s classification, 6 patients (75%) were categorized as Pure PCA, and 2 (25%) as PCA Plus, including PCA‐CBS and PCA‐AD/DLB mixed. Phenotypically, 3 patients were dorsal variant, 3 ventral, and 2 biparietal. CSF AD biomarkers were positive in 2 Pure PCA patients and 1 PCA‐AD/DLB patient. MRI revealed posterior cortical atrophy in all cases. FDG‐PET showed parieto‐temporo‐occipital hypometabolism, with frontal involvement in dorsal/ventral subtypes, while biparietal subtypes showed occipital sparing. Tau‐PET in selected cases demonstrated increased uptake in affected regions, matching FDG‐PET findings.

**Conclusion:**

PCA remains underrecognized due to its atypical presentation and early onset. This series highlights the clinical and imaging heterogeneity of PCA in Indian subjects. Advanced imaging, like FDG/Tau‐PET, can aid in distinguishing phenotypes. Greater research is needed to explore phenotypic variations, identify biomarkers, and develop targeted therapies.